# Exploring Longitudinal Links and Mechanisms Connecting Views on Aging With Health in Later Life

**DOI:** 10.1093/geroni/igaa057.1968

**Published:** 2020-12-16

**Authors:** Allyson Brothers, Susanne Wurm, Liat Ayalon

## Abstract

A robust body of evidence points to the importance of views on aging (VoA) in influencing developmental outcomes in later life. Now that this connection has been well-established, research has turned to examine mechanisms, processes, and pathways by which VoA and health are interconnected throughout adult development. The presentations in this symposium use longitudinal datasets to address the pressing question of how VoA and health are associated over time. Timescales range from 6-months post-hospital stay, to 9 year longitudinal follow-up. Presentations will provide evidence concerning both physical and mental health conditions and symptoms (e.g., Brothers et al; Schönstein et al.). Furthermore, the studies focus on various aging outcomes, ranging from functional ability (Brothers et al.) to recovery from hospital stays (Blawert et al.), to sensory loss (Wettstein et al.), to specific medical conditions including depression, back pain, rheumatism (Schönstein et al.). Three of the talks provide evidence for the effects of VoA on health, and one presents evidence of specific health conditions as longitudinal predictors of later VoA. Thus, an important question that this cohesive set of talks will raise is the issue of bi-directionality, by which VoA affect health, and vice versa. Concluding the symposium, the discussant will share insights about how these presentations contribute to the current understanding of mechanisms of the associations between VoA and health, and the role of VoA for ageism. She will additionally provide an overview of new areas for future research that can help to advance the understanding of these complex interactions.

